# Young Healthy Patient With Severe COVID-19 and Fulminant Community-Acquired Pseudomonas aeruginosa Pneumonia: A Case Report

**DOI:** 10.7759/cureus.32617

**Published:** 2022-12-16

**Authors:** Yusuke Kusaka, Taku Ogawa, Tomoyuki Yamada, Kenta Minami, Osamu Umegaki, Akira Ukimura

**Affiliations:** 1 Department of Anesthesiology, Osaka Medical and Pharmaceutical University, Osaka, JPN; 2 Department of Microbilogy and Infection Control, Osaka Medical and Pharmaceutical University, Osaka, JPN; 3 Infection Control Center, Osaka Medical and Pharmaceutical University, Osaka, JPN

**Keywords:** pneumonia, community-acquired, co-infection, pseudomonas aeruginosa, covid-19

## Abstract

Community-acquired pneumonia (CAP) caused by *Pseudomonas aeruginosa* in healthy adults can rapidly lead to severe outcomes. We treated a case of *P. aeruginosa*-induced CAP and concurrent severe coronavirus disease (COVID-19) in a healthy 39-year-old man without other serious risk factors for severe illness except smoking. Immediately after admission, the patient developed sepsis and received intensive broad-spectrum antibacterial therapy with meropenem and vancomycin, veno-arterial extracorporeal membrane oxygenation (VAECMO), and catecholamine supplementation. Despite receiving multidisciplinary treatment, the patient died within 24 hours. *P. aeruginosa *with normal antimicrobial susceptibility was identified in blood and sputum cultures of samples taken at admission. Gram staining of the bacteria detected in blood cultures was suspicious for non-glucose-fermenting Gram-negative rods, including *P. aeruginosa*, and the antimicrobial regimen that was initiated following admission was considered effective. The patient was a plumber and a smoker, which are risk factors for *P. aeruginosa-*induced CAP, and the clinical course matched those in previous reports of *P. aeruginosa-*induced CAP, including necrotizing pneumonia with cavities and rapid progression of sepsis. Although COVID-19 can be the sole cause of septic shock, the combination of *P. aeruginosa* bacteremia and COVID-19 was possibly the cause of septic shock in this case. Even during an infectious disease pandemic, reviewing the patient's occupational history and comorbidities and performing blood and sputum culture tests, including Gram staining, are important for the provision of appropriate treatment.

## Introduction

The coronavirus disease (COVID-19) is an acute respiratory illness caused by SARS-CoV-2 that emerged in late 2019 and caused a pandemic [1-3]. In Japan, COVID-19 has occurred in six major waves of transmission that have resulted in 7.1 million infections and more than 28,000 deaths. The fourth wave of COVID-19 in Japan was characterized by a younger patient population and more critically ill cases due to the alpha variant, which had increased transmissibility [4]. In addition, many patients in the fourth wave were forced to receive home recuperation due to the lack of availability of critical care beds. In the fourth wave, we experienced a case of COVID-19 infection wherein the patient’s general condition deteriorated during home recuperation due to a community-acquired Pseudomonas aeruginosa infection, which we have reported herein.

## Case presentation

**Figure 1:**
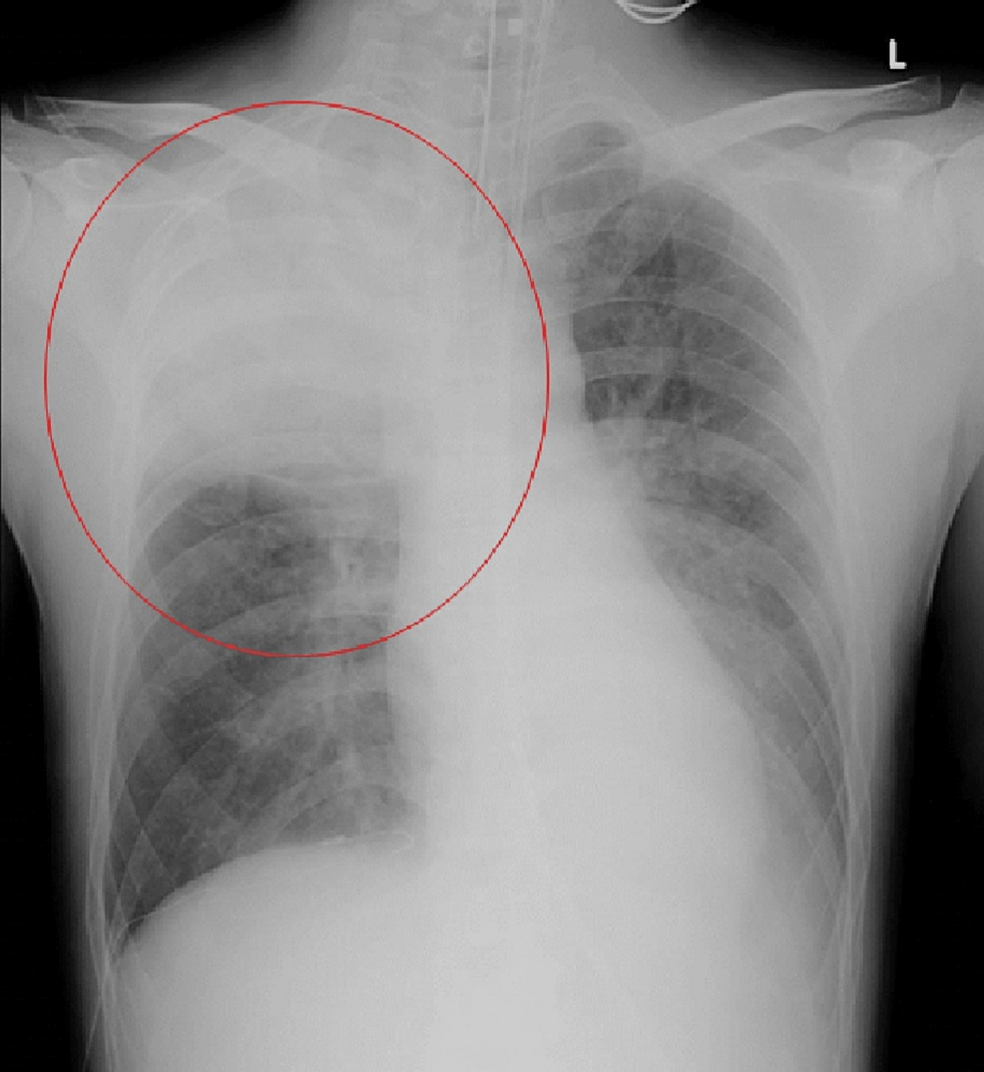
Chest X-ray image at admission indicating a dense infiltrative shadow in the right upper lung field.

**Figure 2:**
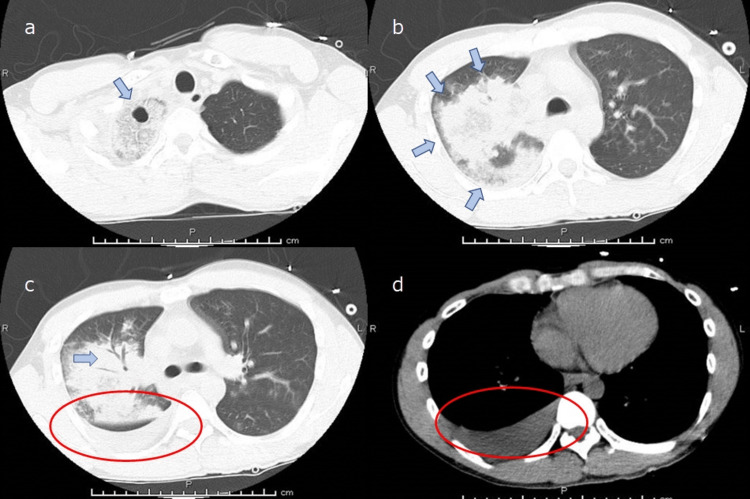
Plain CT image of the chest at admission. Cavity formation is seen in the right upper lobe (indicated by blue arrow in image a). Dense infiltrative shadow is seen throughout the right lung, and air bronchogram is also observed (indicated by blue arrows in images b and c). Right pleural effusion is also observed (indicated by red circles in images c and d).

A 39-year-old man (height 166 cm; weight 52 kg) without significant medical history except for smoking 20 cigarettes a day for 20 years presented to the hospital with a high fever and was diagnosed with COVID-19 after a positive polymerase chain reaction (PCR) test result taken one day after onset. This was the first COVID-19 infection for this patient. He was not vaccinated against COVID-19. This case occurred before COVID-19 vaccinations were initiated for this age group. He continued to receive medical care at home; however, on the eighth day from the onset, he developed a high fever of up to 41 °C and right-sided chest pain without decreased peripheral oxygen saturation (SpO_2_). On the ninth day of his illness, the patient experienced worsening chest pain and respiratory distress and was rushed to the hospital. In the emergency room, clinical examination revealed a patent airway, a respiratory rate of 30 cycles/min, peripheral SpO_2_ of 89% (on 10 L/min oxygen administration using a reservoir mask), a heart rate of 156 beats/min, blood pressure of 88/58 mmHg, the Glasgow Coma Scale at 15 points, and a body temperature of 39.1 °C. Laboratory tests showed elevated leukocyte count (8500/μL) with neutrophilic predominance (88.3%), thrombocytopenia (155,000/μL), elevated creatinine level (4.04 mg/dL), and high levels of inflammatory markers (C-reactive protein 4.84 mg/dL and procalcitonin 12.2 mg/dL). Arterial blood gas analysis showed a pH of 7.080, a base excess of −15.5 mmol/L, and a lactate of 85.5 mg/dL, which indicated severe acidemia due to lactic acidosis. The PO_2_ of the arterial blood was 72.3 mmHg (on 10 L/min oxygen administration using a reservoir mask). The results of blood tests at admission are summarized in Table 1. Chest radiography showed an infiltrating shadow with atelectasis in the right upper lobe. Computed tomography of the chest showed an infiltrating shadow with cavity formation and pleural effusion in the right upper lobe (Figures 1-2). Based on the above-described clinical findings, the patient was diagnosed with COVID-19 and bacterial pneumonia and was admitted to the intensive care unit (ICU) for multidisciplinary treatment. After ICU admission, the patient was immediately intubated, and mechanical ventilation was started. Blood, pleural effusion, and sputum cultures were performed, and empirical antibiotic treatment with meropenem (1.0 g t.i.d.) and vancomycin (25 mg/kg loading dose, followed by 15 mg/kg b.i.d.) was administered. Vasopressor support with noradrenaline (0.15 μg/kg/min) via infusion pump was started, and vasopressin 0.5 U/h was added three hours after admission to maintain circulatory status. Five hours after ICU admission, the patient underwent cardiopulmonary arrest due to severe hypotension and was resuscitated with intravenous adrenaline administration; however, the blood pressure could not be maintained even with a high-dose continuous infusion of vasoconstrictors. Finally, veno-arterial extracorporeal membrane oxygenation (VAECMO) was planned for circulatory support and was initiated by inserting an inlet cannula (21 Fr.) into the right atrium from the femoral vein and an outlet cannula (16 Fr.) into the femoral artery. Initially, VAECMO flow was relatively sufficient; subsequently, however, pulse pressure gradually decreased. Transthoracic echocardiography showed reduced left ventricular (LV) contraction. We requested cardiologists insert an intra-aortic balloon pump (IABP) for reduction of LV afterload. The IABP was successfully inserted from the left femoral artery and activated; however, significant hemodynamic improvement could not be achieved even with the combination of noradrenaline 0.5 µg/kg/min and adrenaline 0.03 µg/kg/min. Finally, the patient died due to peripheral circulatory failure despite various interventions to provide mechanical support. Blood and pleural effusion cultures showed *P. aeruginosa* infection with good antimicrobial susceptibility (Table 2). Written informed consent was obtained from a bereaved family for the submission of this case report to an academic journal.

**Table 1:** Summary of laboratory findings including arterial blood gas analysis. WBC: white blood cells; RBC: red blood cells; HCT: hematocrit; PLT: platelet count; CRP: C-reactive protein; PCT: procalcitonin; TP: total protein; AST: aspartate aminotransferase; ALT: alanine aminotransferase; LDH: lactate dehydrogenase; ALP: alkaline phosphatase; CPK: creatine phosphokinase; BUN: blood urea nitrogen; PT-INR: international normalized ratio of prothrombin time; APTT: activated partial thromboplastin time; FDP: fibrinogen degradation products; BE: base excess.

Test item	Result	
Complete blood count		
WBC	8500	/µL
Neut	88.3	%
Baso	0.7	%
Eosino	0.5	%
Mono	0.7	%
Lymph	9.8	%
RBC	4.66 × 10^6^	/µL
HGB	15.4	mg/dL
HCT	43.8	%
PLT	155 × 10^3^	/µL
CRP	4.84	mg/dL
PCT	12.2	ng/mL
Biochemistry		
TP	5.5	g/dL
Alb	3.4	g/dL
AST	768	U/L
ALT	303	U/L
LDH	986	U/L
ALP	121	U/L
CPK	1097	U/L
BUN	36	mg/dL
Cre	4.04	mg/dL
Na	128	mmol/L
K	3.8	mmol/L
Cl	91	mmol/L
Glu	55	mg/dL
T-bil	1	mg/dL
PCT	12.2	ng/mL
Coagulation factors		
PT-INR	3.53	
APTT	42.6	Sec
FDP	16.1	µg/mL
Arterial blood gas analysis		
pH	7.08	
PCO_2_	50.1	mmHg
PO_2_	72.3	mmHg
$\mathrm{HCO}_{3}^{-}$	14.5	mmol/L
BE	−15.5	mmol/L
Lac	85.5	mg/dL
10L/min with a reservoir mask		

**Table 2:** Results of susceptibility testing of Pseudomonas aeruginosa obtained from the blood culture sample of the patient. MIC: minimal inhibitory concentration.

Pseudomonas aeruginosa		
Antibiotic	MIC (µg/mL)	Interpretive categories
Piperacillin	8	Susceptible
Tazobactam/piperacillin	8	Susceptible
Ceftazidime	2	Susceptible
Cefepime	2	Susceptible
Imipenem	1	Susceptible
Meropenem	≤0.25	Susceptible
Aztreonam	4	Susceptible
Amikacin	≤2	Susceptible
Gentamicin	≤1	Susceptible
Levofloxacin	0.5	Susceptible
Ciprofloxacin	≤0.25	Susceptible

## Discussion

The incidence of *P. aeruginosa* as a causative organism in community-acquired pneumonia (CAP) ranges from 0.9% to 6% [5-6]. CAP caused by *P. aeruginosa* is more common in patients with underlying respiratory disease and is rare in patients with no or a minor underlying disease [7]. Furthermore, *P. aeruginosa* CAP with no or minimal underlying illness has been reported to have a more rapid and intense clinical course [8-10]. Mortality rates range from 28% to 63.1%, with a median time from admission to death of only 11 hours. The most frequently reported clinical manifestations in the acute phase include chest pain, a dry cough, and hemoptysis, which often lead rapidly to septic shock [10-12].

Moreover, many reports indicate that most CAP caused by *P. aeruginosa* results in necrotizing pneumonia and that the foci often appear in the right upper lobe. However, the reason for this manifestation is unclear [12]. It is estimated that 92% of these cases are associated with bacteremia [12], and the collection of blood and sputum cultures is crucial. The case we reported was of a patient with overlapping CAP and severe COVID-19, and the rapid deterioration of the general condition could have been explained by COVID-19 alone. However, we could ascertain the pathophysiology by performing a sputum examination and blood culture tests on samples obtained at admission. Unfortunately, by the time we had established the patient's condition, septic shock, probably caused by *P. aeruginosa* bacteremia, had already progressed, and we could not save the patient’s life. This case occurred in the middle of the fourth wave of COVID-19 (with the alpha variant) in Japan when access to hospitals was more difficult than usual. It is unknown if this fact is attributive to the patient's prognosis. However, it may be necessary to make the medical system in Japan more efficient so that patients with pneumonia do not remain without hospitalization for nine days.

Although CAP due to *P. aeruginosa* is rare, several risk factors have been reported. First is using aerosol-producing devices such as humidifiers, home whirlpool spas, and hot tub bathing [13-14]. Occupation-related risk factors have been reported to include being a nursing assistant and working in an elderly care facility [8,15]. Dust-contaminated metals have also been reported to be associated with *P. aeruginosa* pneumonia [16], and there have been reports of *P. aeruginosa* CAP in healthy young men who worked in areas where aerosolized contaminated metalworking fluid with P. aeruginosa was present [10]. In addition, although there is no direct data for *P. aeruginosa* pneumonia, it has been reported that Gram-negative rods, including *P. aeruginosa*, tend to be more prevalent among oral commensal bacteria in smokers [17]. This patient was a plumber, and it is assumed that he routinely worked in an environment where aerosols contaminated with bacteria, particularly *P. aeruginosa*, were present. In addition, the patient's smoking habit may have put him at higher risk for CAP caused by *P. aeruginosa*.

As mentioned above, limited access to medical care due to the COVID-19 pandemic, which led to limited access to hospitals, might be the primary cause of the poor prognosis of this patient. Nevertheless, what we could have done differently if he had been seen in the hospital before deteriorating to the point of ICU admission is also unclear. Risk factors for CAP caused by *P. aeruginosa* include antimicrobial therapy within the past three months, underlying respiratory diseases such as cystic fibrosis, bronchiectasis, chronic obstructive pulmonary disease with repeated exacerbations, diabetes mellitus, or alcohol abuse. The patient we experienced, in this case, was a young, healthy adult without any of the abovementioned underlying diseases. From the perspective of rational antimicrobial use, it is difficult to justify the initiation of an antipseudomonal antibiotic based only on COVID-19 history in this patient. Although there are not many studies on the frequency of complications between COVID-19 and bacterial pneumonia, a study by Langford et al. reported the following result [17,18]: bacterial complications accounted for 6.9% of all COVID-19 patients or 5 out of 34 cases in which the causative bacterial organism was identified. This value is high compared to common community-acquired pneumonia, in which *P. aeruginosa* accounts for 2.2-2.9% of cases in the report of Shoar et al. [19]. However, in the study by Langford et al., there was no clear information on whether the patients had been admitted to an elderly care facility or hospitalized prior to the onset of COVID-19; therefore, we may not necessarily conclude that P. aeruginosa is the most common causative organism of bacterial pneumonia associated with COVID-19. Certainly, there is not much we could have done had this case been admitted a little earlier. However, if he had been admitted at least before oxygenation had worsened, we would have been able to observe the Gram-stained sputum smear and carefully follow up with the patient in case of sudden deterioration. As a result, the prognosis might have been different.

## Conclusions

We experienced a case of fulminant *P. aeruginosa* pneumonia complicated by COVID-19 in a young male with no underlying disease. Although *P. aeruginosa* is a rare cause of CAP, it should not be forgotten as a differential diagnosis. The possibility that *P. aeruginosa* is the causative organism should be considered, especially in COVID-19 cases who present with necrotizing pneumonia with cavity formation, have an occupation with daily exposure to aerosols, or have a smoking habit.
